# Preserving the Pelvic Ring at the Sciatic Notch During Resection of Malignant Bone Tumors at the Posterior Ilium

**DOI:** 10.1111/os.12783

**Published:** 2020-10-11

**Authors:** Akio Sakamoto, Bungo Otsuki, Shimei Tanida, Shunsuke Fujibayashi, Shuichi Matsuda

**Affiliations:** ^1^ Department of Orthopaedic Surgery, Graduate School of Medicine Kyoto University Kyoto Japan

**Keywords:** Bone tumor, Ilium, Pelvis, Sacroiliac joint, Surgery

## Abstract

Resection of malignant bone tumors in the posterior ilium may result in pelvic ring disruption. Preserving the pelvic ring and keeping an adequate surgical margin is ideal, but is challenging, especially when the tumor extends to the sacroiliac joint. The current report proposes a line from the lateral point of the second sacral dorsal foramen to the anterior surface of sacral ala (S_2_‐sacral ala line), and cutting from the line to the ilium over the sciatic notch and to the sacral wing using thread saws. This preserves the cortex at the sciatic notch and the distal sacroiliac joint. Two posterior iliac tumors extending to the sacroiliac joint, a metastatic melanoma in a 75‐year‐old male, and an osteosarcoma in a 56‐year‐old male were resected. The resections were performed along the S_2_‐sacral ala line, and consequently lumbo‐sacro‐pelvic fusions were performed. Both patients were able to walk with one crutch. Indications for the method using the S_2_‐sacral ala line for iliac tumors may be limited. However, the method can increase pelvic ring preservation in cases with posterior iliac malignant bone tumors.

## Graphical abstract

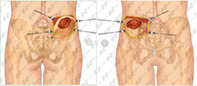

Preserving the pelvic ring at the sciatic notch during resection of a malignant bone tumor (brown) at the posterior ilium, with a resection line (red dotted line) and S_2_‐sacral ala points (green dot) of the lateral point of the second sacral dorsal foramen (S_2_ point: left illustration) and the anterior surface of sacral ala (sacral ala point: right illustration).

## Introduction

The importance of maintaining the stability of the pelvic ring is demonstrated by the favorable functional results that we report in these cases after partial resection, rather than complete resections with reconstruction^1^, ^2^. Preserving the pelvic ring during tumor resection can achieve better function with fewer postoperative complications, particularly infection^3^. Three anatomical zone classifications are commonly used for resection of pelvic bone tumors^4^. The ilium is a common site for pelvic bone tumors^5^, and resection of the ilium bone tumor, with or without partial sacral resection, is classified as P1^4^. Preserving the pelvic ring during resection of a malignant bone tumor in the posterior ilium is challenging, particularly when it extends to the sacroiliac joint. The difficulty derives from the anatomical complexity, even in cases in which the tumor has not extended to the sciatic notch or the distal sacroiliac joint. In this report, positive outcomes are demonstrated for malignant bone tumors at the posterior ilium when the S_2_‐sacral ala line is used to preserve the pelvic ring at the sciatic notch during resection. The goal of the study is to describe the details of the surgical procedure by providing representative cases.

## Methods

### *Operative Procedure*

#### *Exposure of the Sciatic Notch*

**Fig. 1:**
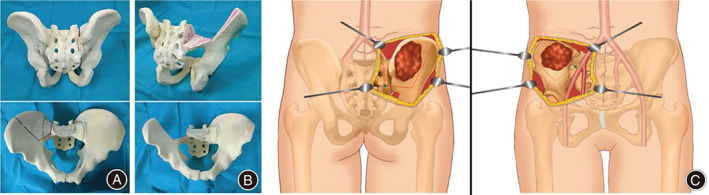
A saw bone model for pelvic ring preservation surgery. Views before (A) and after (B) the cutting. Oblique views show preservation of the pelvic ring at the sciatic notch and distal sacroiliac joint (B). Illustrative diagrams of posterior (C‐left) and anterior (C‐right) views, showing a tumor (brown) with a resection line (red dotted line) and S_2_‐sacral ala points (green dot) of the lateral point of the second sacral dorsal foramen (S_2_‐point: C‐left) and the anterior surface of sacral ala (sacral ala‐point: C‐right).

This method is indicated for bone tumors located at the posterior ilium that have extended to the sacroiliac joint, but not the sciatic notch or the distal sacroiliac joint. A saw bone model (Fig. 1A,B) and an illustrative diagram (Fig. 1C) are shown in Fig. 1. The surgeries were performed under general anesthesia with the patient in a lateral position. An incision was made from the back with a vertical portion on the sacroiliac joint, along the right iliac crest, to the superior anterior iliac spine. During resection of the malignant bone tumor at the posterior ilium, the iliac crest and the sacrum are exposed, allowing for tumor‐free margins around the tumor, which may extend into the soft tissue. The iliac muscle was sectioned or detached from the endopelvic side of the ilium, and the sciatic notch was exposed by detaching the gluteal muscles from the ilium.

#### *Cutting the Ilium Above the Sciatic Notch*

The lateral point of the second sacral dorsal foramen was exposed (S_2_ point) above the sciatic notch over the inferior–posterior iliac process. The cut line was designed to be <10 mm above the edge of the sciatic notch and extend to the iliac crest according to the resection plan. Next, the medial side of the ilium at the retroperitoneum was exposed, and to the anterior surface of sacral ala at the resection line was confirmed (Fig. 1C). A Kirschner wire was inserted from the S_2_ point to the anterior surface of sacral ala (S_2_‐sacral ala line) or from the opposite direction. A navigation system was used to confirm the S_2_ point and the anterior surface of sacral ala point, if necessary (VectorVision II; BrainLAB, Munich, Germany). However, skilled surgeons may be able to insert a Kirschner wire from the anterior surface of sacral ala point to the S_2_ point without this assistance. A thread saw was inserted through the S_2_‐sacral ala line; and a steel cannulated needle, such as an inner cannulated needle for bone biopsy (TSK Laboratory, Japan, 13 G, 100 mm Surelock), can be a useful guide. From S_2_‐sacral ala line, the ilium over the sciatic notch to the ilium was cut with a thread saw according to the preoperative plan. Cutting the outer cortex of the ilium with a high‐speed burr or a small bone saw helped to make an accurate cut with the thread saw.

#### *Cutting the Sacral Wing*

After cutting the ilium, the sacral wing was cut from S_2_‐sacral ala line with another thread saw. Before cutting the sacral wing, the surface of the sacral wing was exposed, while protecting the fifth sacral root. Subsequently, lumbo‐sacro‐pelvic fusion was performed with screws and rods. Full weight‐bearing was allowed after the operation.

## Case Reports

The method is indicated for bone tumors located at the posterior ilium with extension to the sacroiliac joint but without extension to the sciatic notch or the distal sacroiliac joint. In the current report, two cases treated using this method are presented. One case is a 75‐year‐old male with a malignant melanoma in the right ilium, and the other was a 56‐year‐old male with an osteosarcoma in the left ilium. Informed consent was obtained from the patients to present the current report.

### *Case 1: Malignant Melanoma, Metastatic*

**Fig. 2:**
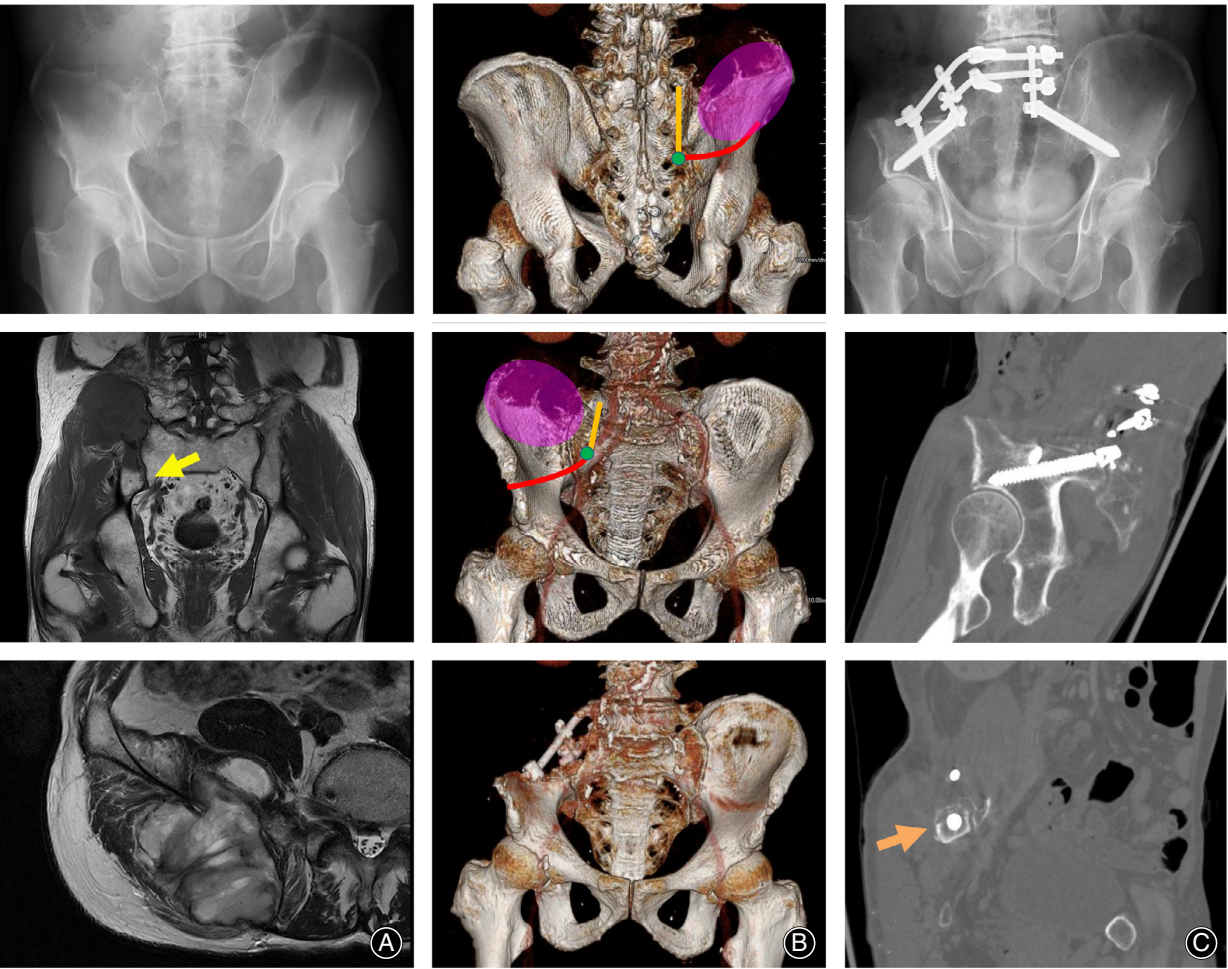
Metastatic melanoma in the ilium of a 75‐year‐old male. A plain radiograph shows an osteolytic lesion of the right posterior ilium (A‐top). An MRI shows low signal intensity on T1‐weighted images (A‐middle) and a heterogeneous signal intensity on a T2‐weighted image (A‐bottom). No tumor extension is seen at the distal ilium (A‐middle; a yellow arrow). Schematics of the tumor (pink) with resection lines from the iliosacral joint to ilium (red line) and sacrum (orange line), and the S_2_‐sacral ala point (green dot) are shown on CT with three‐dimensional reconstruction (B‐top and B‐middle). CT with three‐dimensional reconstruction (B‐bottom) and A plain radiograph (C‐top) 12 months after resection and lumbo‐sacro‐pelvic fusion are shown. The CT shows the sciatic notch in longitudinal (C‐middle) and transverse (C‐bottom; an orange arrow) views.

A 75‐year‐old male noticed a growing right iliac mass 5 months before the initial assessment. At the initial assessment, a 10‐cm elastic hard mass was palpable. Tenderness or redness over the lesion was not observed. Plain radiographs showed an osteolytic lesion at the posterior ilium adjacent to the sacroiliac joint (Fig. 2A). Magnetic resonance imaging (MRI) showed a lesion with homogenous iso‐signal intensity to the muscle on T1‐weighted images, with heterogeneous low‐to‐high signal intensity on a T2‐weighted image with a nodular appearance (Fig. 2A). ^18^F‐fluorodeoxyglucose positron emission tomography/computed tomography (FDG‐PET/CT) depicted a solitary lesion with a maximum standardized uptake value (SUVmax) of 26.3. The lesion extended to the upper part of the sacroiliac joint, although it did not appear to spread to the sciatic notch or distal sacroiliac joint (Fig. 2A). Histology from a needle biopsy showed a malignant tumor, for which no specific classification was assigned.

The tumor was resected with a surgical margin using S_2_‐sacral ala line for reference (Fig. 2B). The defect was filled with autologous graft, which was fixed with u‐HA/PLLA plates and screws. A lumbo‐sacro‐pelvic fusion was performed with rods and screws (Fig. 2C). Full weight‐bearing was allowed after the operation. Histologically, the resected material was used to diagnose a malignant melanoma. Although no primary lesion was detected, the bone lesion was considered to be a metastatic (stage IV) melanoma of unknown primary (MUP) according to the Cancer Staging Manual of the American Joint Committee on Cancer (AJCC) staging system. Because tumor invasion was suspected at the surgical margin microscopically, radiation therapy (60 Gy in 30 fractions at 2.0 Gy per fraction) was delivered. Subsequently, chemotherapy of intravenous pembrolizumab was administered. The patient was able to walk with a crutch without pain medications but exhibited a Trendelenburg gait due to insufficient strength of the gluteus medius muscle. No recurrence of the tumor was observed up to 1.5 years after the operation.

### *Case 2: Osteosarcoma*

**Fig. 3:**
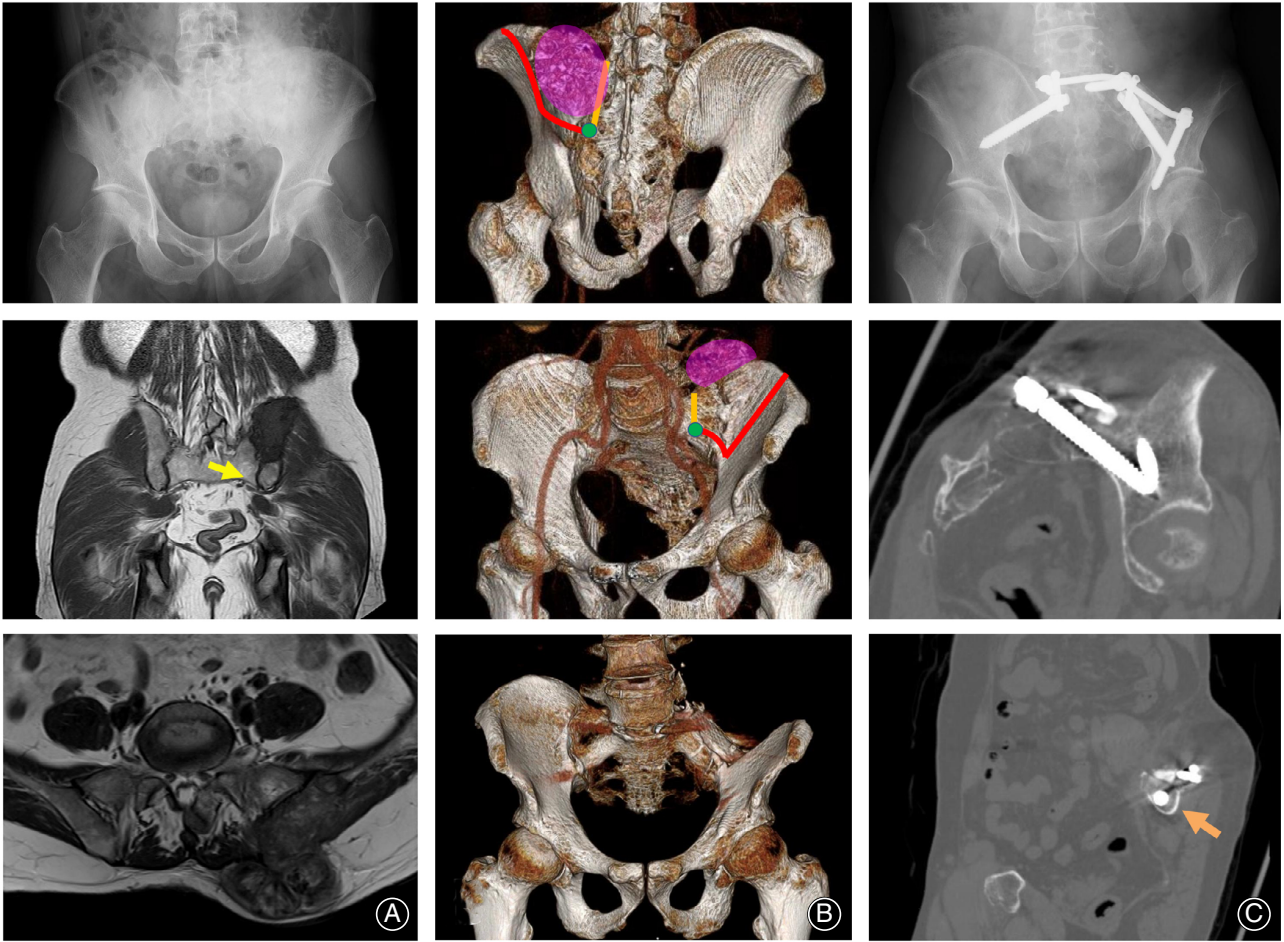
Osteosarcoma in the ilium of a 56‐year‐old male. A plain radiograph shows a neoplastic bone formation at the left posterior ilium (A‐top). An MRI shows low signal intensity on T1‐weighted images (A‐middle) and a heterogeneous signal intensity on a T2‐weighted image (A‐bottom). No tumor extension appears at the distal ilium (A‐middle; a yellow arrow). Schematics of the tumor (pink) with resection lines from the iliosacral joint to ilium (red line) and sacrum (orange line), and (S_2_‐sacral ala point (green dot) are shown CT with three‐dimensional reconstruction (B‐top and B‐middle). CT with three‐dimensional reconstruction (B‐bottom) and a plain radiograph (C‐top) 7 months after resection and lumbo‐sacro‐pelvic fusion are shown. The CT shows the sciatic notch in longitudinal (C‐middle) and transverse (C‐bottom; an orange arrow) views.

A 56‐year‐old male noticed a left iliac mass 3 years before the initial assessment. The size of the lesion had gradually increased. After visits to a nearby hospital, the patient was referred to our institution. Tenderness over the lesion was not reported. Plain radiographs showed a calcified lesion at the posterior ilium (Fig. 3A). An MRI showed a 7‐cm diameter lesion with homogeneous low signal intensity on T1‐weighted images and with heterogeneous low‐to‐intermediate signal intensity on a T2‐weighted image (Fig. 3A). The abnormal signal intensity extended to the sacral wing and normal intensity was maintained at the distal sacroiliac joint (Fig. 3A). The FDG‐PET/CT showed a solitary lesion that was not metastatic and the SUVmax was 7.4. The histological sample from a needle biopsy showed a neoplastic osseous lesion, which suggested it was osteosarcoma.

Resection was prioritized rather than the administration of preoperative chemotherapy. After resection with a surgical margin using S_2_‐sacral ala line for reference (Fig. 3B), the defect was filled with β‐TCP blocks, which were fixed with u‐HA/PLLA plates and screws. A lumbo‐sacro‐pelvic fusion was performed with rods and screws (Fig. 3C). The resected sample was diagnosed as stage IIB (T2, N0, M0, G3) osteosarcoma according to the AJCC staging system. The surgical margin of the resected material was negative for sarcoma. Consecutive chemotherapy with ifosfamide, etoposide, and carboplatin was performed for three courses. The patient was able to walk with a crutch without pain medication but exhibited a Trendelenburg gait. No recurrence was seen 1 year after the operation.

## Discussion

The insertion point for S_2_‐alar screws is lateral, ranging from the midpoint between the S_1_/S_2_ dorsal foramina and the S_1_ dorsal foramen^8^, ^9^. The insertion point using the S_2_‐sacral ala line is more distal to that of S_2_‐alar screws, because the S_2_‐alar screw is directed toward the acetabulum, while the cut line, which follows the S_2_‐sacral ala line, is designed to be almost parallel to the cortex at the sciatic notch^10^.

Navigation systems have been developed to assess accurate screw insertion for spine surgery based on preoperative or intraoperative CT imaging^11^, ^12^, ^13^. In our cases, screw insertions for the lumbo‐sacro‐pelvic fusions were performed using a navigation guide. With respect to resection of the tumor, extraosseous extension of the iliac tumor makes it difficult to expose the bone surface, and a navigation system can be helpful to understand the anatomical reorganization caused by the tumor. In making the S_2_‐sacral ala line, a navigation system was used to confirm the position of the S_2_ dorsal foramen and the anterior surface of sacral ala. However, adequate identification of these anatomical landmarks is easy for a skilled surgeon who can identify them without a navigation system, by using fluoroscopy.

In summary, using a line from the lateral point of the second sacral dorsal foramen to the anterior surface of sacral ala (S_2_‐sacral ala line) can preserve the cortex at the sciatic notch and distal sacroiliac joint during resection of a posterior iliac tumor. The indication for this method may be limited. However, the method can increase pelvic ring preservation in some cases, which previously has been difficult to achieve.
